# Removal of Protein Capping Enhances the Antibacterial Efficiency of Biosynthesized Silver Nanoparticles

**DOI:** 10.1371/journal.pone.0134337

**Published:** 2015-07-30

**Authors:** Navin Jain, Arpit Bhargava, Mohit Rathi, R. Venkataramana Dilip, Jitendra Panwar

**Affiliations:** Centre for Biotechnology, Department of Biological Sciences, Birla Institute of Technology and Science, Pilani, 333 031, India; VIT University, INDIA

## Abstract

The present study demonstrates an economical and environmental affable approach for the synthesis of “protein-capped” silver nanoparticles in aqueous solvent system. A variety of standard techniques viz. UV-visible spectroscopy, transmission electron microscopy (TEM), energy dispersive spectroscopy (EDS) and X-ray diffraction (XRD) measurements were employed to characterize the shape, size and composition of nanoparticles. The synthesized nanoparticles were found to be homogenous, spherical, mono-dispersed and covered with multi-layered protein shell. In order to prepare bare silver nanoparticles, the protein shell was removed from biogenic nanoparticles as confirmed by UV-visible spectroscopy, FTIR and photoluminescence analysis. Subsequently, the antibacterial efficacy of protein-capped and bare silver nanoparticles was compared by bacterial growth rate and minimum inhibitory concentration assay. The results revealed that bare nanoparticles were more effective as compared to the protein-capped silver nanoparticles with varying antibacterial potential against the tested Gram positive and negative bacterial species. Mechanistic studies based on ROS generation and membrane damage suggested that protein-capped and bare silver nanoparticles demonstrate distinct mode of action. These findings were strengthened by the TEM imaging along with silver ion release measurements using inductively coupled plasma atomic emission spectroscopy (ICP-AES). In conclusion, our results illustrate that presence of protein shell on silver nanoparticles can decrease their bactericidal effects. These findings open new avenues for surface modifications of nanoparticles to modulate and enhance their functional properties.

## Introduction

Over the past few years, worldwide escalation and augmentation of multi-drug resistance in microorganisms has been a serious concern for modern medicine [1–5]. The need for the development of new, low cost and effective antimicrobial agents independent of bacterialresistance has revived the interest of scientific community to explore the antimicrobial properties of silver and its compounds. In past few years, variety of new silver formulations such as silver sulfadiazine, silver citrate, silver lactate, etc. have been developed and various silver integrated formulations (Katadyn, Argyrol, Movidyn, Tetrasil, Alagon, etc.) were commercialized [6]. However, cost concern and recent reports of silver-resistant bacterial strains has limited the use of silver as a potential alternative to antibiotics [7].

Since, most of the biological processes take place at the nanoscale level, a combined application of biology and nanotechnology can perhaps meet this challenge [8]. Utilization of silver nanoparticles can be particularly advantageous as compared to their bulk counterpart as the former manifest high surface area to volume ratio which can provide better contact with microorganisms. It has been demonstrated that silver nanoparticles restrict the microorganisms to develop resistance [9–11]. Additionally, at low concentrations silver nanoparticles have been reported to be non-toxic to human cells, and hence considered as an safe antimicrobial agent [12]. Owing to these properties, an enormous increase in the applications of silver nanoparticles for a wide range of medical and commercial products has been observed which includes household antiseptic sprays and antimicrobial coatings for medical devices that sterilze air and surfaces [13]. Silver nanoparticles have also expanded their horizons in textiles, cosmetics, air purifiers, food packaging, coating for refrigerators, water disinfection; in fact, in every application where bacteria may exert a harmful effect [14].

The mechanism of bactericidal effects of silver nanoparticles has not been yet completely elucidated. In particular, there is a debate as to whether the toxicity is only due to the silver nanoparticles or it is confined to the silver ions released from these nanoparticles. It has been reported that the release of silver ions from the crystalline core of silver nanoparticles contribute to the bactericidal effects [15]. In aerobic conditions, silver nanoparticles get oxidised releasing high concentrations of silver ions in solution, which interacts with proteins giving rise to bactericidal effects [16]. The antibacterial activity of micromolar concentration of silver ions may be linked with uncoupling of respiratory electron transport from oxidative phosphorylation [17], inhibition of respiratory chain enzymes and interference with the membrane permeability [18] or interaction with cytoplasmic components and nucleic acids [19]. However, the molecular mechanism behind the bactericidal activity of silver ions is still not clear. In addition, whether the bactericidal effects are exerted by nanoscale phnemenon is an elusive question. Xiu et al. [20] attempted to decipher this challenge by performing antibacterial studies under strict anaerobic conditions to preclude silver oxidation and Ag^+^ release. Their findings completely ruled out the direct particle-specific bactericidal effects of silver nanoparticles and inferred Ag^+^ ions as the definitive molecular toxicant for bactericidal effects. Thus, a strict control over the release of silver ions is a pre-requisite for the antibacterial efficacy of silver nanoparticles. Manipulation at various levels such as particle size, morphology, surface charge, coating and oxygen availablity have been considered as important parameters to control and modulate the antibacterial activity of silver nanoparticles. Among these parameters, surface coating (or functionalization) serve as the most important factor which determine the nanoparticle-microbe interactions. It has been observed that coating of silver nanoparticles with surfactants results in greater damage to microorganisms in comparison to similar-sized bare silver nanoparticles [21,22]. Similarly, comparitive studies of polysaccharide coated and bare silver nanoparticles showed that presence of polysaccharide molecules on nanoparticle surface facilitates the damage to mammalian cell-lines [23]. Conversely, nanoparticles were found to be less toxic to microorganisms when coated with polymers or natural organic matter [24]. Variegated effects of surface chemistry on silver nanoparticle uptake and toxicity have also been reported. For instance, presence of molecules such as albumin, lecithin, polysorbital-80 and peptide on the nanoparticle surface have been reported to facilitate their uptake and toxicity, while polyethylene glycol interfered with uptake in the liver cells [25]. In contrast, a systematic sub-chronic reproduction toxicity assessment of silver nanoparticles coated with polyvinylpyrrolidone (hydrophilic) or oleic acid (amphiphilic) on soil earthworms (*Eisenia fetida*) showed no significant differences in silver accumulation or toxicity [26]. Hence, studies targeted to understand the dynamic behaviour of nanoparticle coatings (inorganic or organic) could be highly informative for designing efficient antibacterial formulations of silver nanoparticles.

In the present study, we are reporting a one-step protocol to synthesize “protein-capped” silver nanoparticles using *Aspergillus* sp. NJP02. The presence of protein shell on nanoparticles is highly advantageous as it imparts solubility and colloidal stability in aqueous system. In order to find out the role of protein shell in modulating antibacterial efficacy, the comparative antibacterial potential of protein-capped and bare silver nanoparticles were assessed. Our results clearly indicate that the presence of protein shell over the surface of silver nanoparticles negatively affect their antibacterial potential. Further mechanistic studies suggested that protein-capped and bare silver nanoparticles demonstrate distinct mode of action.

## Experimental

Fungus *Aspergillus* sp. NJP02 (GenBank accession number: HM222932) was used for the extracellular synthesis of silver nanoparticles. The complete details for isolation and molecular characterization of the isolate can be obtained from our previous report [27].

### Synthesis and characterization of protein-capped and bare silver nanoparticles

The extracellular synthesis of silver nanoparticles was achieved using an indigenous protocol developed in our laboratory [28]. Briefly, the fungus was grown in MGYP medium for 72 h at 28°C on a rotary shaker (150 rpm) under dark conditions. After incubation, the fungal mycelia were separated and washed thrice with sterile water in order to remove all traces of media. Typically, 10 g of biomass (fresh weight) was resuspended in 100 mL of sterile deionized Milli-Q water and further incubated for 72 h under the same conditions as described above. After incubation, biomass was separated by filtration using Whatman filter paper no. 1 and the fungal cell free filtrate containing extracellular secretions was collected. For synthesis of silver nanoparticles, aqueous silver nitrate solution at a final concentration of 1.0 mM was added to the reaction vessels containing cell-free filtrate and incubated at 28°C on a rotary shaker (150 rpm) without light. The obtained protein-capped silver nanoparticles were used for further experiments. The particles were characterized by UV-visible spectroscopy, dynamic light scattering (DLS), transmission electron microscopy (TEM), energy dispersive spectroscopy (EDS) and selected area electron diffraction (SAED) analysis. The detailed procedures for the above analyses have been discussed in our previous report [28]. The crystalline phase of nanoparticles was measured by X-ray diffraction (XRD) studies using a Rigaku MiniFlex-II bench top diffractometer operated at a voltage of 40 kV and current of 30 mA with CuK_α_ radiation. In order to check the stability of protein-capped silver nanoparticles, UV-visible spectrum of three month old sample was also recorded.

For preparation of bare silver nanoparticles, the as-synthesized protein-capped silver nanoparticle solution was centrifuged at 10,000 rpm for 20 min. The pellet was suspended in 1% (w/v) sodium dodecyl sulphate (SDS) and boiled in water bath for 30 min in order to detach the protein shell from nanoparticles followed by centrifugation at 10,000 rpm for 20 min. The supernatant containing the unreacted SDS and SDS-protein complex was analyzed for the presence of proteins by measuring the UV-visible absorption spectrum. The resulting pellet was boiled in 1 mL of Tris-Cl (pH 8.0) in water bath for 10 min to eliminate the possibility of SDS binding to the nanoparticles, if any. To ensure the complete removal of SDS, dialysis was carried out against Milli-Q water with four changes of water. The obtained bare silver nanoparticles were characterized using Fourier transform infrared spectroscopy (FTIR), Photoluminescence (PL) spectroscopy, UV-visible spectroscopy and DLS measurements. FTIR spectra of freeze-dried samples were recorded on a Shimazdu IR Prestige-21 FTIR spectrometer. Photoluminescence measurements were performed on a Horiba FluroMax-4 spectrofluorometer with an excitation wavelength of 280 nm using 90° illumination. In order to achieve maximal signal-to-noise ratio, excitation and emission slit width values were attuned to 2.5 and 3.0 nm, respectively. UV-visible spectroscopy measurements were carried out on a Jasco V-630 UV-visible spectrophotometer at a resolution of 1 nm. DLS measurements were conducted using a Malvern Zetasizer Nano ZS instrument and the obtained data were analysed using Zetasizer software.

### Antibacterial efficiency of silver nanoparticles

*In vitro* bactericidal effects of protein-capped and bare silver nanoparticles were evaluated against clinically-important bacterial pathogens procured from the Institute of Microbial Technology, India. The tested strains were Gram positive *Bacillus cereus* (MTCC 430) and *Pseudomonas putida* (MTCC 102); and Gram negative *Escherichia coli* (MTCC 1652) and *Klebsiella pneumoniae* (MTCC 432). The selected bacterial species were exposed to protein-capped or bare silver nanoparticles (50 μg nanoparticles per mL of medium) for 30 min at 37°C unless otherwise stated.

#### Assays for antibacterial activity

To examine the effect on bacterial growth rate, the selected bacterial species were grown separately in 100 mL of nutrient broth supplemented with 50 μg of protein-capped or bare silver nanoparticles per mL of medium. The bacterial growth rates were determined by measuring the absorbance at 600 nm at different time intervals (0.1 absorbance corresponds to a concentration of 10^8^ cells per mL). Media without nanoparticles and bacterial cells were used as positive and negative controls, respectively.

Minimum inhibitory concentration (MIC) values of protein-capped and bare silver nanoparticles were determined by performing dehydrogenase assay in a 96-well plate. Bacterial inoculums were prepared by washing the overnight grown culture twice with phosphate buffer saline (pH 7.4) followed by dilution to achieve a final concentration of 10^8^ cfu mL^-1^. 100 μL of bacterial inoculum and 20 μL of triphenyl tetrazolium chloride (TTC; 3 mg mL^-1^ solution) were added in each well. Subsequently, 20 μL of 50 μg mL^-1^ silver nanoparticle (protein-capped or bare) solution was added and the plates were incubated for 18 hours at 37°C under dark conditions. The triphenyl formazan (TPF) formed was measured at 485 nm using an ELISA reader. The MIC assay was done in triplicate and executed thrice to validate the MIC values for the each tested bacterial species.

#### Assays for antibacterial mechanism

The generation of intracellular reactive oxygen species (ROS) in presence of protein-capped or bare silver nanoparticles was determined using an oxidation-sensitive fluorescent dye 2,7-dichlorodihydrofluorescein diacetate (DCFH-DA) in triplicate as per the standard protocol [29]. Briefly, the overnight grown bacterial cells (10^8^ cfu mL^-1^) were washed thrice with phosphate-buffered saline (pH 7.4) and further suspended in fresh nutrient broth. DCFH-DA (10 μM in phosphate-buffered saline) was mixed in the medium at a ratio of 1:2,000 (dye: medium) and incubated for 30 min at 37°C in dark conditions to successfully penetrate the dye into the bacterial cells. The DCFH-DA loaded cells were separated from the free dye molecules by centrifugation at 8,000 rpm for 5 min followed by a final wash with the phosphate-buffered saline (pH 7.4). The bacterial pellets were exposed to protein-capped or bare silver nanoparticles as mentioned earlier. In living bacterial cells, intracellular esterases result in hydrolysis of DCFH-DA to non-fluorescent 2,7-dichlorodihydrofluorescin (DCFH) which in presence of ROS oxidizes to fluorescent dichlorodihydrofluorescin (DCF) [30]. The fluorescent signal intensity of DCF was measured by Perkin Elmer VICTOR X Multilabel Plate Reader at an excitation and emission wavelength of 485 nm and 535 nm, respectively. Bacterial cells incubated in 25 mM ascorbic acid for 1 h before DCFH-DA exposure were used as positive control while cells without DCFH-DA treatment were used as negative control [31].

Peroxidase and superoxide dismutase (SOD) activities were measured in triplicate following the method of Hochman and Goldberg [32] and Arora et al. [33], respectively. After exposure, the bacterial cells were centrifuged at 8,000 rpm for 5 min and the pellet was sonicated to obtain the crude enzyme extract. Bacterial culture without nanoparticle treatment served as control.

Reaction mixture for the assay of peroxidase contained 1 mL of 0.01 M pyrogallol, 2 mL of 0.1 M phosphate buffer (pH 6.0), and 1 mL of 5 mM hydrogen peroxide [32]. The reaction was initiated by adding 1 mL of enzyme extract and the mixture was incubated at 25°C for 5 min. Subsequently, the reaction was terminated by adding 1 mL of 2.5 N H_2_SO_4_ and the amount of purpurogallin formed was estimated by measuring the absorbance at 420 nm. In blank, 1 mL of sterile water was used instead of extract. One unit of peroxidase was defined as the amount of enzyme required to form 1 mg of purpurogallin per min under the specified conditions.

The SOD assay is based on the ability of SOD to inhibit the photochemical reduction of nitroblue tetrazolium (NBT) [33]. Reaction mixture for the assay of SOD contained 13.0 mM methionine, 6.3 μM NBT, 6.5 μM riboflavin, 0.1 mM EDTA, and 50 mM phosphate buffer (pH 7.8). The reaction was initiated by adding 500 μl enzyme extract (diluted 10 times) to 1.5 mL of reaction mixture followed by incubation at 30°C for 10 min under 6000 lux light intensity. After incubation, the tubes were immediately transferred to dark conditions and the absorbance was measured at 560 nm. Reaction mixture containing sterile water instead of enzyme extract served as blank. The non-irradiated reaction mixture served as negative control. One unit of the SOD activity was defined as the amount of enzyme required to inhibit the reduction of NBT by 50% under the specified conditions.

Malondialdehyde (MDA) formation, a measure of membrane damage was monitored using thiobarbituric acid (TBA) as a probe molecule [34]. After exposure to protein-capped or bare silver nanoparticles, the bacterial cells were hydrated in 1 mL of 2.5% (w/v) trichloroacetic acid (TCA) and subsequently centrifuged at 12,000 rpm for 20 min at 4°C. The supernatant (100 μl) was mixed with 0.5% (w/v) TBA reagent prepared in 20% (w/v) TCA. The reaction mixture was heated at 100°C for 30 min in a water bath followed by centrifugation at 12,000 rpm for 10 min at 4°C. The absorbance of the MDA-TBA adduct was measured at 532 nm. MDA content was expressed as picomoles per mg of protein using a molar extinction coefficient of 1.56 mM^-1^ cm^-1^ [34]. The extent of membrane leakage was also determined by quantification of protein [35], total sugar [36] and nucleic acid content. The nucleic acid leakage was detected by measuring the absorbance of culture supernatant at 260 nm (1 OD unit = 50 μg mL^-1^ nucleic acid).

#### Inductively coupled plasma atomic emission spectroscopy (ICP-AES) measurements

The dissolution of silver ions from protein-capped or bare silver nanoparticles was measured in nutrient medium after exposure to bacterial cells. The silver ions were separated by dialyzing the culture medium against sterile Milli-Q water for 12 h using a 12 kDa cellulose membrane and measured by Shimadzu ICPS-7500 instrument. The silver ion concentration in nutrient medium (without silver nanoparticles) was used as control.

#### TEM imaging

In order to prepare sample for TEM imaging, nanoparticle treated bacterial cells were centrifuged at 5,000 rpm for 2 min. The bacterial pellets were washed twice with phosphate buffer saline (pH 7.2) followed by a pre-fixation step with 2.5% glutaraldehyde (prepared in 0.1 M cacodylate buffer) for 2 h at 4°C. Post-fixation of the bacterial cells was performed with 1% OsO_4_ treatment for 1 h at 4°C. After post-fixation, the OsO_4_ solution was removed by washing twice the bacterial cells with 0.1 M cacodylate buffer followed by staining with filtered uranyl acetate (1%) solution. For TEM imaging, a drop of stained bacterial sample was placed on a carbon-coated copper grid and dried overnight in a vacuum desiccator. The grids were observed on a Hitachi H-7650 TEM instrument operated at a constant voltage of 100 kV.

## Results and Discussion

### Characterization of protein-capped and bare silver nanoparticles

**Fig 1:**
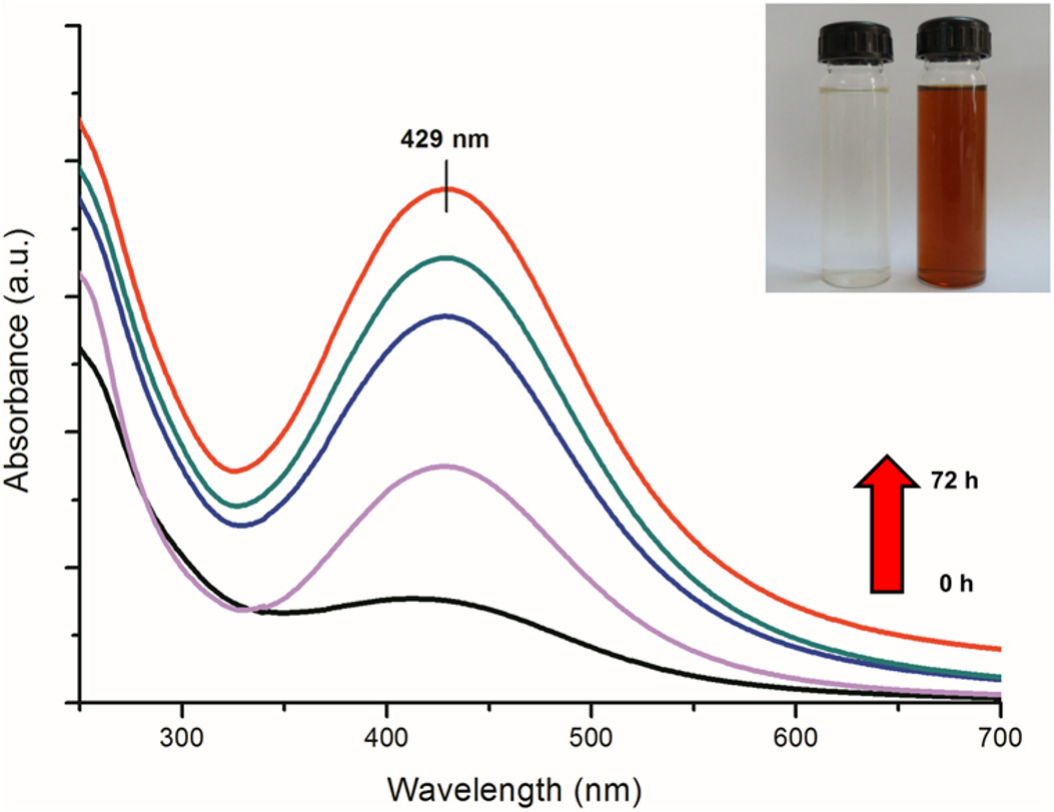
UV visible spectrum of reaction medium as a function of time (0, 12, 24, 48 and 72 h). Inset shows tubes containing fungal cell-free filtrate (a) without and (b) with silver nitrate solution after 72 h of reaction.

A gradual change in the colour of reaction medium (containing fungal cell-free filtrate and precursor silver ions) from colourless to reddish brown revealed a visual evidence for silver nanoparticle synthesis (Fig 1 inset). UV-visible spectrum showed a gradual increase in the absorbance at 429 nm with respect to time (Fig 1). The absorption maxima at 429 nm can be attributed to the surface plasmon resonance (SPR) vibrations of synthesized silver nanoparticles [37]. No further increase in absorbance was observed after 72 h of reaction (data not shown), which indicated the complete reduction of precursor silver ions in reaction medium. Stability of as-synthesized silver nanoparticles was monitored periodically for more than three months. It was observed that the nanoparticle solution was extremely stable at room temperature, with no evidence of particle aggregation as determined by UV-visible spectroscopy measurements.

**Fig 2:**
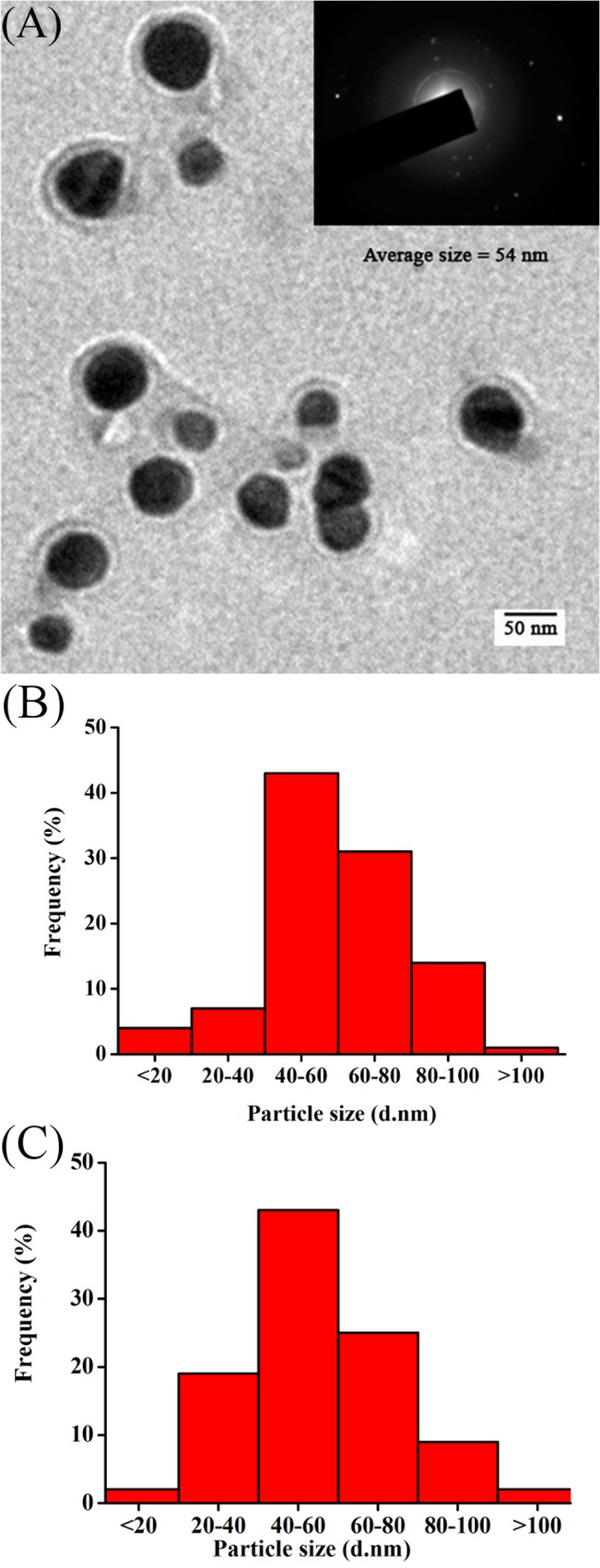
(A) A representative transmission electron micrograph showing spherical shaped silver nanoparticles (scale bar equivalent to 50 nm). Inset showing SAED pattern recorded from a single nanoparticle. Particle size distribution histogram of silver nanoparticles as determined using (B) transmission electron microscope and (C) dynamic light scattering measurements.

TEM micrograph (Fig 2A) revealed the presence of mono-dispersed and predominantly spherical particles with no visible aggregation. The SAED pattern (Fig 2A inset) attests the crystallanity of silver nanoparticles. The particle size distribution histogram obtained from TEM measurements revealed that most of the particles ranged between 40–80 nm with a mean diameter of 54 ± 8.9 nm (Fig 2B). These results were in well agreement with the values obtained by DLS measurements (Fig 2C). It has been well reported that different fungi can synthesize nanoparticles of varied composition, sizes and shapes which may be due to the differences in their extracellular protein profiles [38–40].

**Fig 3:**
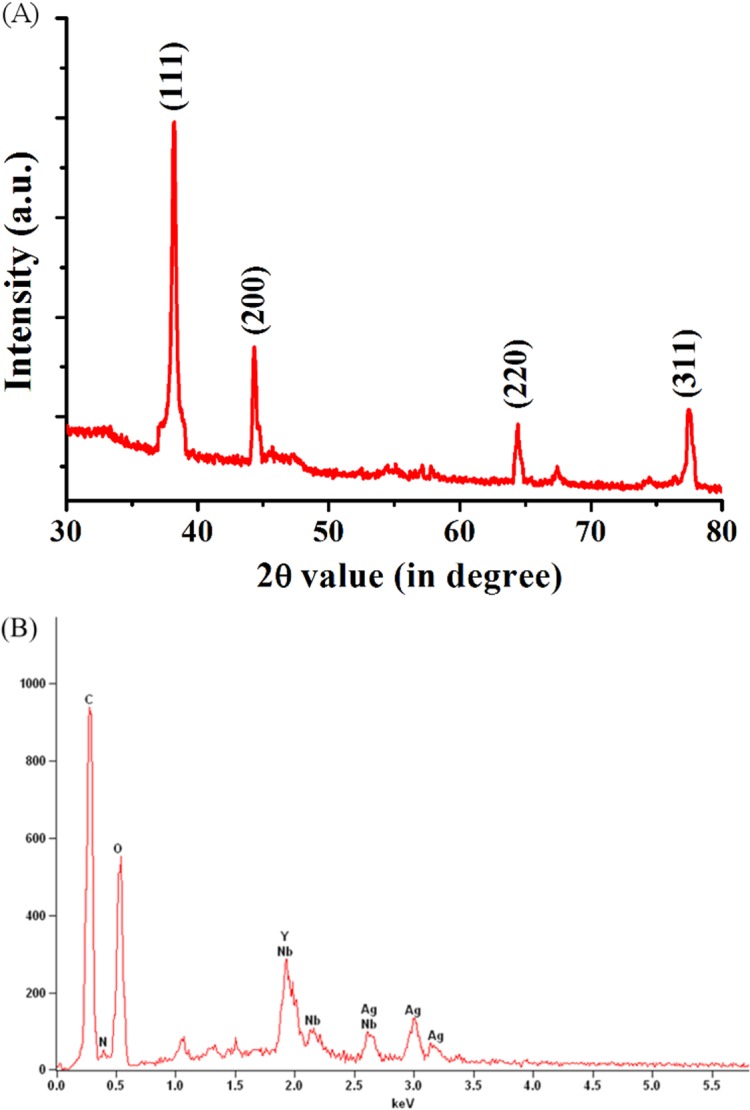
(A) XRD spectrum of as-synthesized protein-capped silver nanoparticles with Bragg’s diffraction values shown in parentheses. (B) EDS spectrum showing the elemental composition of silver nanoparticles.

X-ray diffraction pattern recorded by preparing drop-coated film of protein-capped silver nanoparticles further validated the crystalline nature of nanoparticles. The well-defined peaks at 2θ values of 38.03°, 46.18°, 64.60°, and 77.18° corresponds to (111), (200), (220) and (311) planes of silver, respectively (Fig 3A). These values were in complete agreement with the face-centered cubic (fcc) lattice structure of crystalline silver (JCPDS file no. 04–0783). A similar pattern of XRD spectrum has been reported for metallic silver nanoparticles synthesized by other fungi [41]. EDS was carried out to determine the elemental composition of as-synthesized nanoparticles and capping molecules. An intense optical absorption band at 3.0 KeV confirmed the presence of pure metallic silver nanoparticles (Fig 3B). Other peaks observed for C, N and O atoms indicated the presence of proteins as capping molecules.

**Fig 4:**
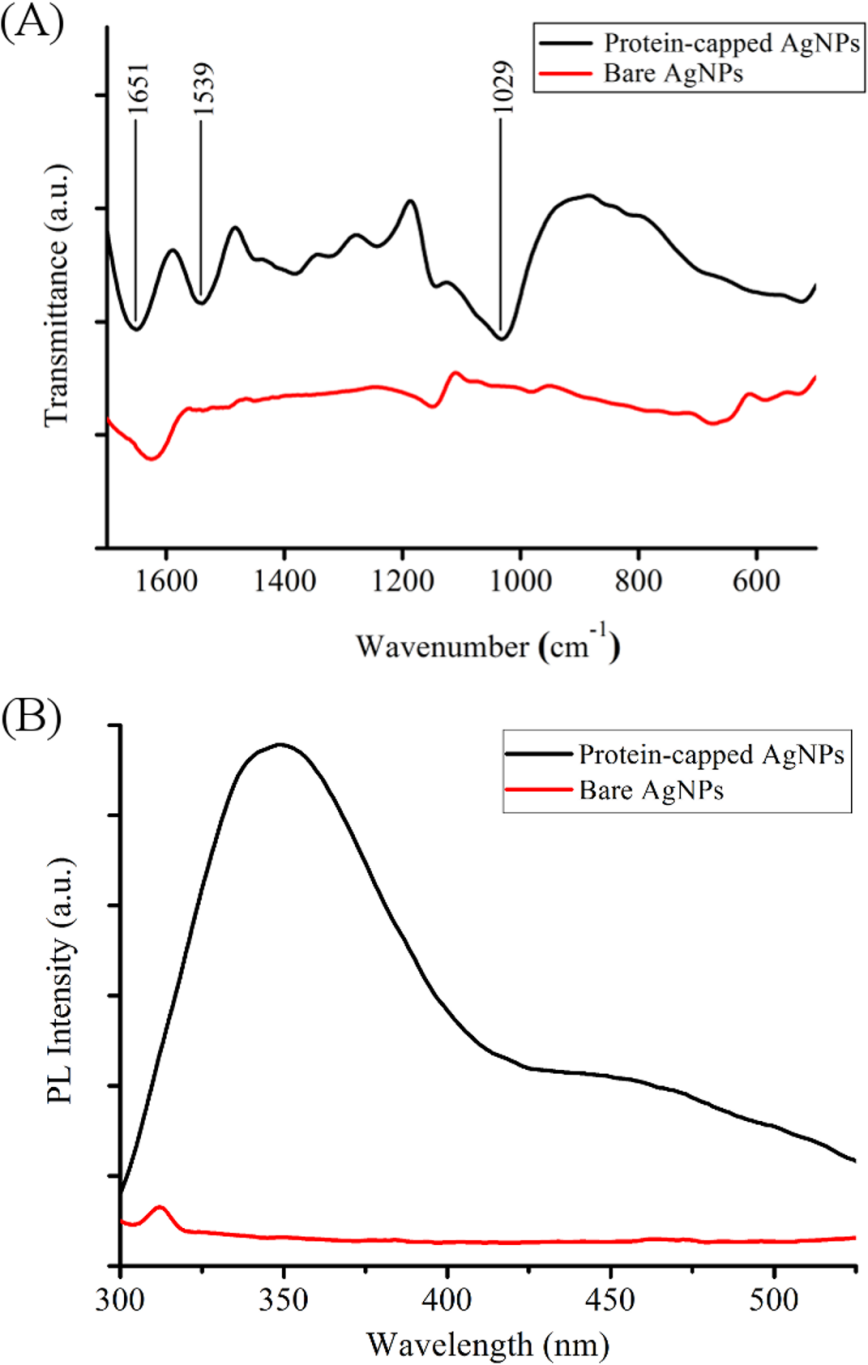
(A) FTIR spectra and (B) Photoluminescence spectra of protein-capped and bare silver nanoparticles.

UV-visible absorption spectrum of supernatant obtained after SDS treatment of as synthesized silver nanoparticles showed a broad absorption peak near 280 nm which corresponds to aromatic amino acids of proteins S1 Fig [28]. FTIR and photoluminescence measurements of protein-capped and bare silver nanoparticles were carried out to confirm the removal of capping proteins from the surface of silver nanoparticles. FTIR spectrum of protein-capped silver nanoparticles (Fig 4A) exhibited characteristic bands of amide I and amide II at wavenumbers 1651 and 1539 cm^-1^, respectively and C-N stretching vibration band of aliphatic amine at 1029 cm^-1^ [28]. The disappearance of these characteristic protein bands in the FTIR spectrum of bare silver nanoparticles clearly indicated the removal of proteins from nanoparticles. The PL spectrum (Fig 4B) of protein-capped silver nanoparticles showed a distinct emission peak at 340 nm, which could be attributed to the tyrosine residues of capping proteins. In contrast, absence of emission peak in case of bare silver nanoparticles confirmed the complete removal of protein molecules.

**Fig 5:**
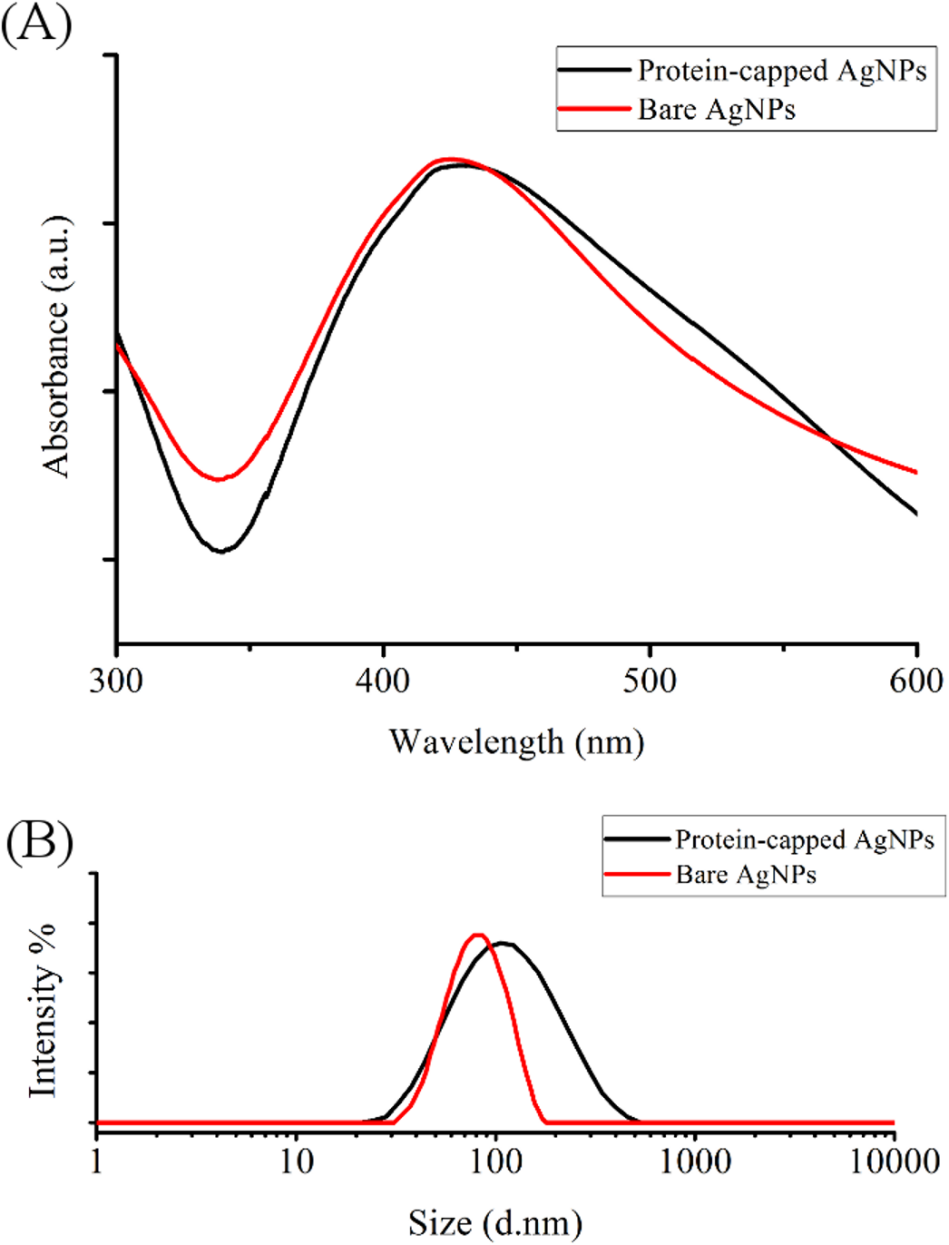
(A) UV visible spectra and (B) particle size distribution of protein-capped and bare silver nanoparticles.

The removal of protein shell by SDS treatment reduced the size of nanoparticles as reflected by the blue shift observed in the SPR peak from 429 to 425 nm in case of bare silver nanoparticle (Fig 5A). Calzolai et al. [42] reported a similar observation while studying interaction between human ubiquitin and gold nanoparticles. Furthermore, hydrodynamic particle size distribution and polydispersity index (PDI) analysis was carried out to investigate the occupancy of protein shell in case of protein-capped silver nanoparticles. The protein-capped silver nanoparticles showed a mean particle size of 90.53 nm (PDI = 0.357) which decreased to 58.39 nm (PDI = 0.396) in case of bare silver nanoparticles indicating the successful removal of protein shell from nanoparticles after SDS treatment (Fig 5B). The plausible reason for multi-layered shell (~32 nm thick) could be the non-specific and non-competitive binding of proteins present in the surrounding environment (reaction medium). The persistence of thick protein shell on biogenic silver nanoparticles has attracted our attention to compare the antibacterial efficacy of protein-capped nanoparticles in comparison to bare silver nanoparticles.

### Antibacterial efficacy of protein-capped and bare silver nanoparticles

The time-dependent bacterial growth in presence of protein-capped and bare silver nanoparticles was monitored by measuring the absorbance at 600 nm [43]. The protein-capped as well as bare silver nanoparticles were found to adversely affect the bacterial growth with major implications on the exponential phase S2 Fig. Growth dynamics profile of both Gram positive bacterial species revealed that the protein-capped nanoparticles were more effective in controlling the bacterial growth as compared to bare silver nanoparticles. In contrast, bare nanoparticles were found to be more effective than protein-capped nanoparticles in case of Gram negative bacterial species. The greater efficacy of bare silver nanoparticles against Gram negative bacteria observed during the present study is in complete agreement with previous reports [19,44,45]. Overall, the impact of both types of silver nanoparticles was observed to be more prominent on Gram negative bacteria as compared to Gram positive bacteria. This can be hypothesized in terms of their different cell wall composition wherein a relatively thick and continuous peptidoglycan cell wall in Gram positive bacteria could restrict the entry of bare silver nanoparticles. However, the interactions of teichoic acid (which span the peptidoglycan layer) and side chains of amino acids of protein-capped silver nanoparticles may facilitate their possible entry in Gram positive bacterial species.

**Fig 6:**
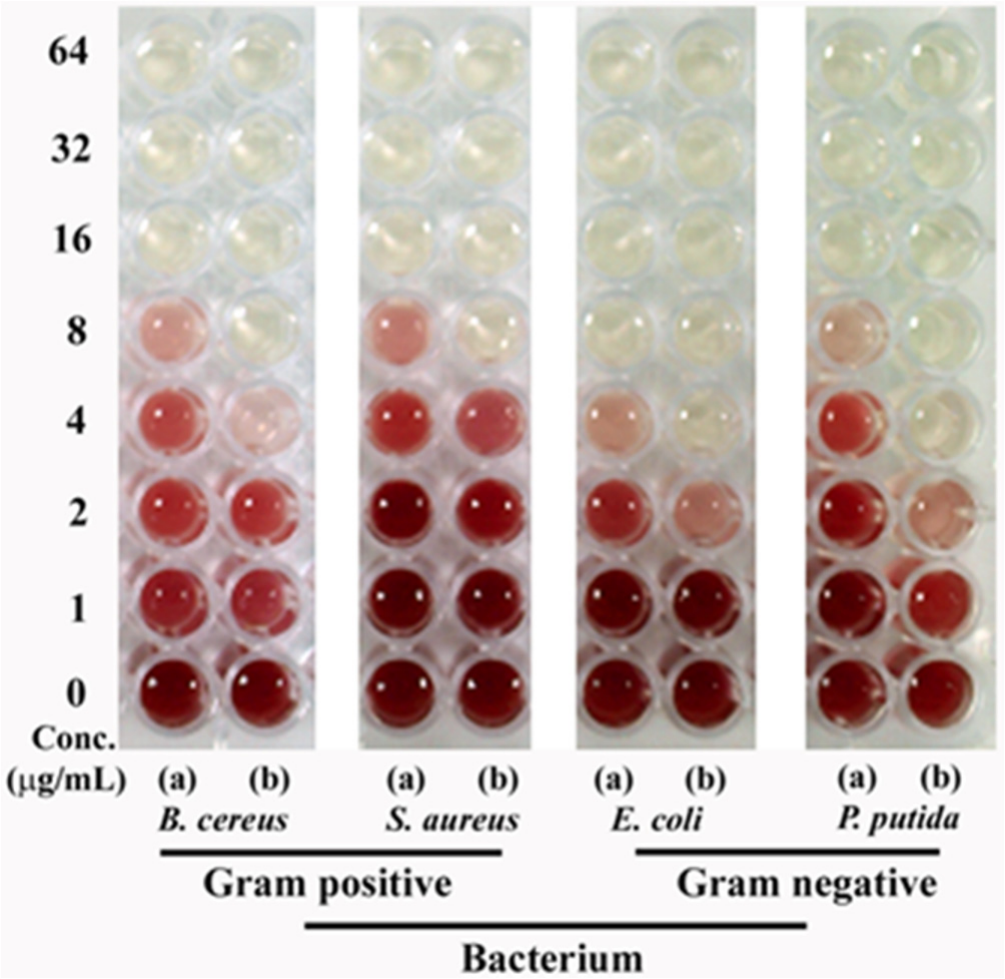
Dehydrogenase assay demonstrating MIC profiles of (a) protein-capped and (b) bare silver nanoparticles against selected Gram positive and Gram negative bacteria.

Based on the doubling time and cell size each bacterial species shows relatively different growth kinetics. Keeping this fact in mind we performed the more compelling minimum inhibitory concentration (MIC) test by dehydrogenase assay. A positive correlation was observed between the antibacterial activity and concentration of nanoparticles (Fig 6). Overall, the tested silver nanoparticles were found to be more effective against Gram negative bacteria as compared to Gram positive bacteria. The observed MIC values for protein-capped silver nanoparticles were 8 μg mL^-1^ and 4–8 μg mL^-1^ for Gram positive and Gram negative bacteria, respectively. On the other hand, MIC values for bare silver nanoparticles were found to be 2–4 μg mL^-1^ and 2 μg mL^-1^ for Gram positive and Gram negative bacteria, respectively. The obtained MIC values suggested that protein-capped silver nanoparticles were less effective as compared to bare silver nanoparticles. This can be attributed to the presence of protein shell on silver nanoparticles. It can be anticipated that the subtle changes on nanoparticle surface with protein shell can modulate their interactions with the bacterial surface, leading to differential antibacterial profiles. To date, however, there is no clear understanding that surface modification of nanoparticles with protein shell can control the antibacterial properties of silver nanoparticles. The modulation of antibacterial profile due to the presence of proteins on nanoparticle made it more interesting and motivated us to decipher the involved mechanism.

### Bactericidal mechanism of protein-capped and bare silver nanoparticles

**Fig 7:**
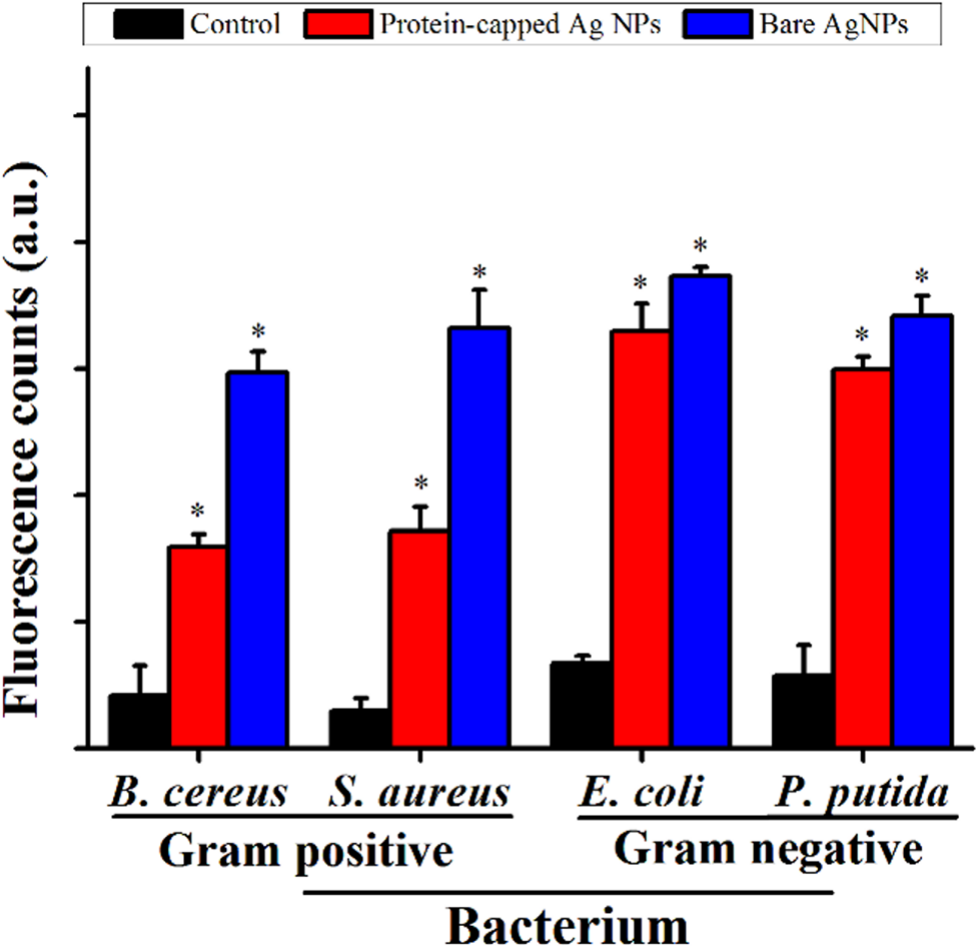
Relative fluorescence intensity (with respect to H_2_O_2_) showing the cellular ROS formation capability of protein-capped and bare silver nanoparticles as compare to control. Vertical bars represent standard errors. Significant differences from control (p ≤ 0.05) are marked with asterisk.

The exact mechanism of bactericidal effect of silver nanoparticles has yet not been elucidated and is open for debate [7,46]. The most commonly reported mechanism is the generation of free radicals such as peroxide, superoxide and hydroxyl ions by silver nanoparticles which could play a key role in executing bactericidal effects [47]. Fig 7 depicts the magnitude of ROS species formation represented as fluorescent counts due to DCF formation in bacterial cells exposed to the protein-capped and bare silver nanoparticles as compared to the control. The magnitude of ROS species generation was approximately two fold higher for bare silver nanoparticles as compared to the protein-capped nanoparticles in case of Gram positive bacterial species. The low ROS production observed in case of protein-capped silver nanoparticles may be attributed to the presence of biocompatible molecules in the shell. In contrast, absence of these biocompatible molecules on bare silver nanoparticles led to accelerated ROS production. Surprisingly, almost similar magnitude of ROS species generation was observed for both protein-capped and bare silver nanoparticles in Gram negative bacterial species.

**Fig 8:**
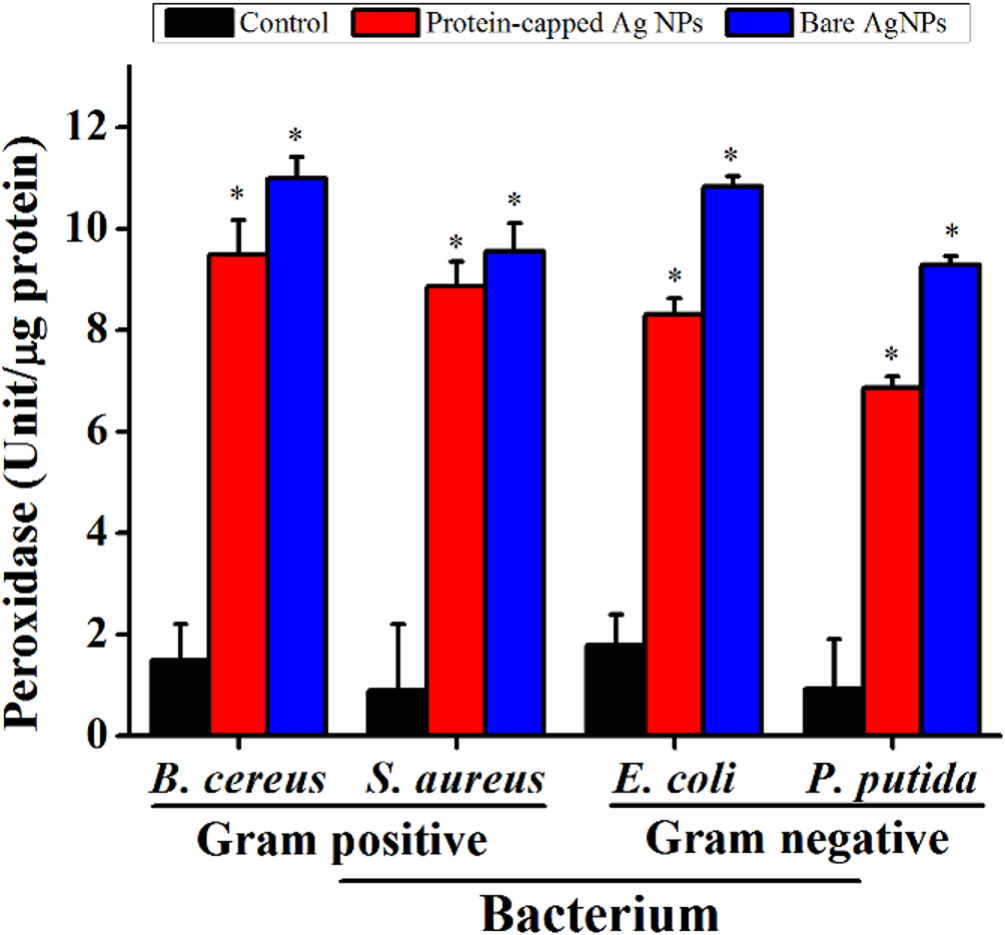
Levels of peroxidase in untreated and treated bacterial cells. The data are expressed as mean ± standard error of three independent experiments (p<0.05).

**Fig 9:**
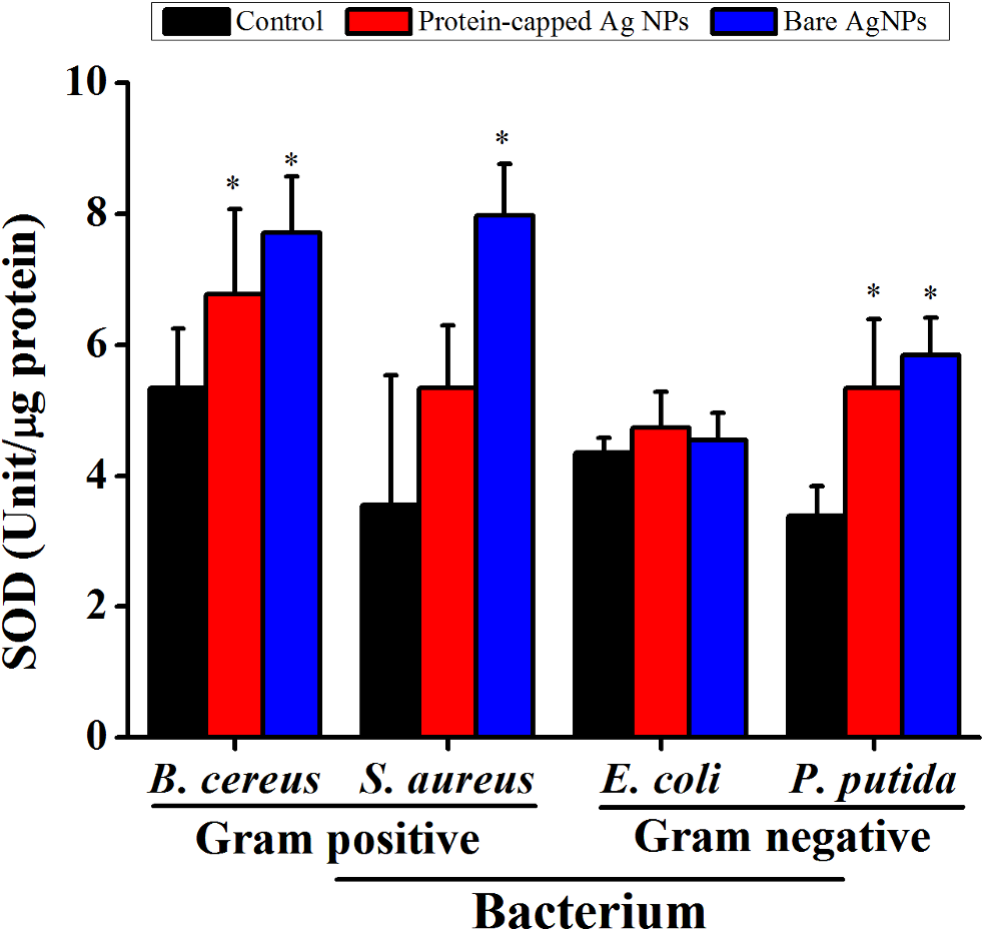
Superoxide dismutase activity in untreated and treated bacterial cells. The data are expressed as mean ± standard error of three independent experiments (p<0.05).

It has well been reported that silver ions catalyse the decomposition of H_2_O_2_ and subsequently results in the formation of free radicals (OH and/or O_2_^-^) [48]. Hence, the role of ROS generation and its consequences on the antioxidant system was evaluated by measuring the activity of ROS related enzymes viz. peroxidase (Fig 8) and SOD (Fig 9). In general, an increase in the activities of both these enzymes was observed in all the tested bacterial species when exposed to bare silver nanoparticles. It can be inferred that the removal of protein shell not only generates higher amount of ROS molecules but also leads to the formation of cellular oxidants which also interfere with the respective antioxidant system [49].

**Fig 10:**
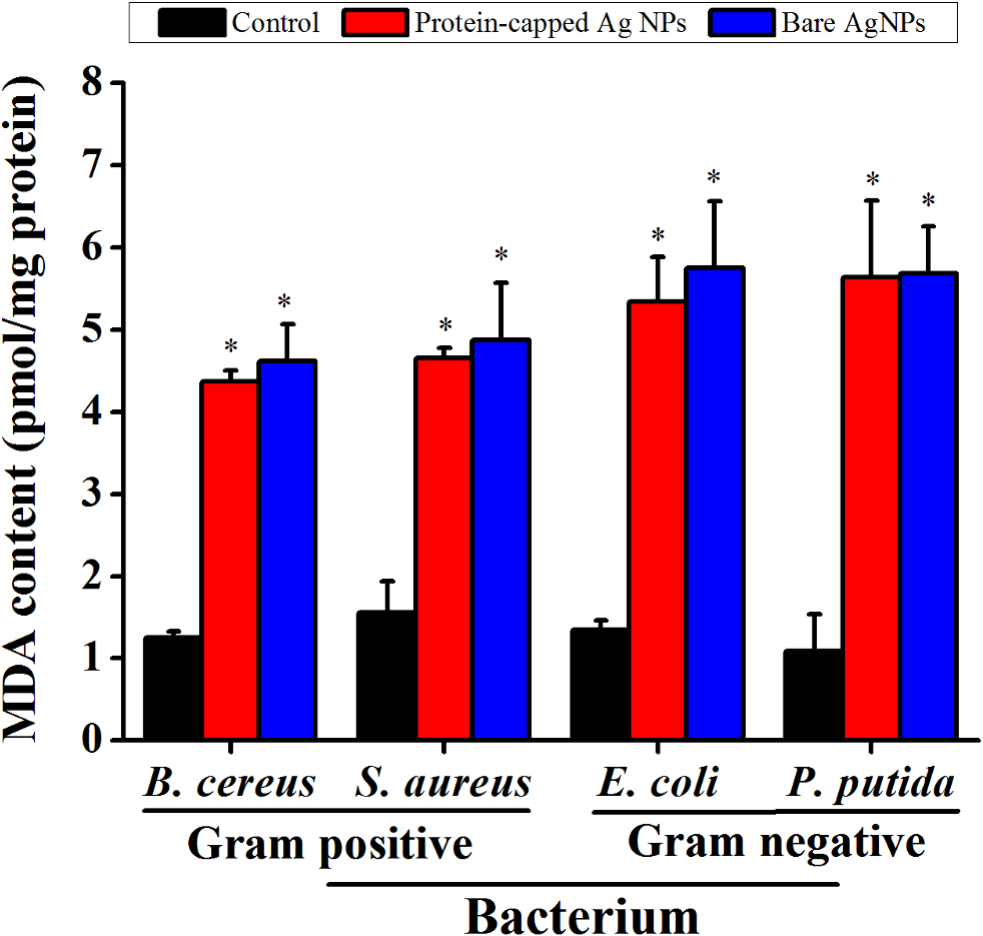
Malondialdehyde (MDA) assay demonstrating the difference in membrane damage capability of protein-capped and bare silver nanoparticles. Vertical bars represent standard errors. Significant differences from control (p ≤ 0.05) are marked with asterisk.

The generation of free radicals such as peroxide, superoxide and hydroxyl ions by silver nanoparticles have been reported to exert lipid peroxidation and damage membrane integrity [9,50–52]. Furthermore, biochemical and proteomic studies strengthened the fact that silver nanoparticles resulted in an immediate dissipation of the proton motive force which causes de-energization of cell membrane and consequently cell death [53]. Keeping these facts in mind, the membrane damage studies were performed by measuring MDA content. In general, an increased MDA content was observed in all the tested bacterial species when exposed to silver nanoparticles (Fig 10). However, marginal difference was observed between protein-capped and bare silver nanoparticles.

To corroborate the membrane damage results, we further estimated the release of bio-molecules (protein, carbohydrate and nucleic acid) from bacterial cells after silver nanoparticle treatment. As expected, a higher membrane leakage was observed in the bacterial cells treated with bare silver nanoparticles as compared to those treated with protein-capped silver nanoparticles S1 Table. Moreover, it was clearly evident that the bactericidal action of silver nanoparticles was more effective against Gram negative bacterial species. Damage to the cell membrane led to cell distortion causing release of carbohydrates, proteins and nucleic acids has also been reported previously [54,55].

**Fig 11:**
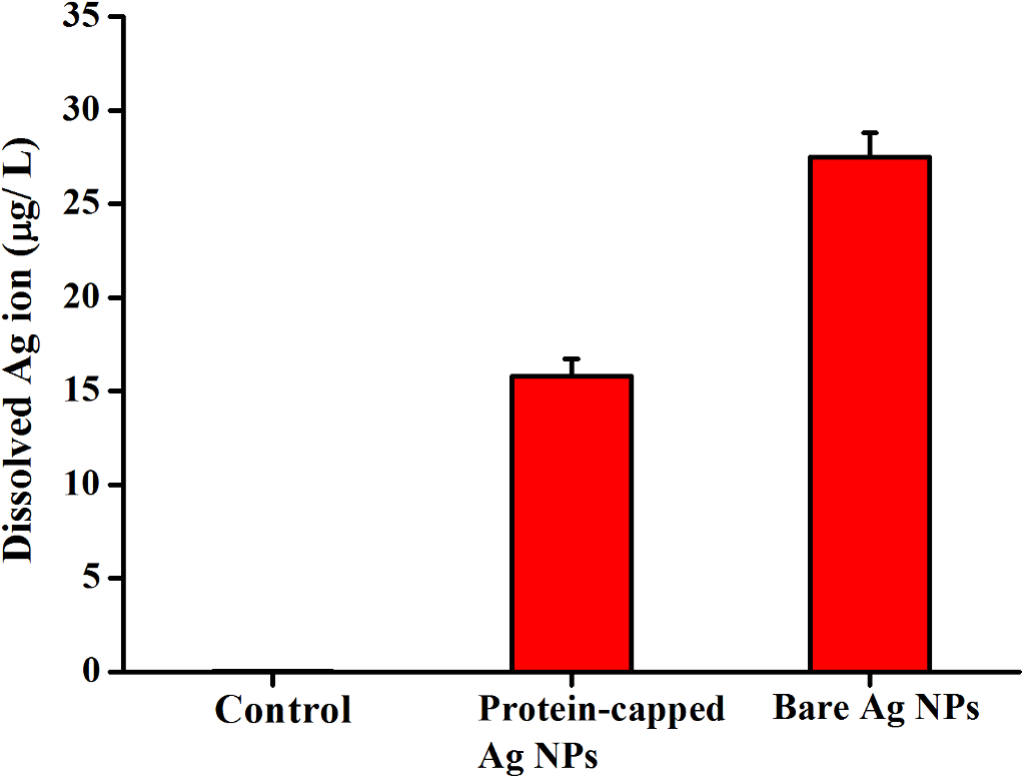
ICP-AES analysis of silver dissolution profiles of protein-capped and bare silver nanoparticles.

The release of silver ions from silver nanoparticles determines their antibacterial activity [46,47,56–58]. It has been demonstrated that aerobic conditions can readily oxidized silver nanoparticles in aqueous solutions resulting in the release of silver ions [59]. The positive charge generated due to release of Ag^+^ ions from silver nanoparticles develops an electrostatic interaction with the negatively charged bacterial cell membrane [60]. Moreover, the reaction of silver ions with carbohydrates, hydroxyls and thiols of bacterial cell wall and nuclear membrane leads to cell distortion and death [12,61]. In the present study, the release of silver ions from protein-capped and bare silver nanoparticles was estimated using ICP-AES. The dissolved silver ion concentration for protein-capped and bare silver nanoparticles was measured as 15.8 and 27.5 μg L^-1^, respectively (Fig 11). It clearly indicates that the presence of protein shell acts as a barrier preventing the release of silver ions from the nanoparticles. These results are in complete accordance with a previous study which demonstrated that surface coating play a key role in dissolution of silver ions from nanoparticle surface and the toxicity of silver nanoparticles depends on the surface coating [62].

**Fig 12:**
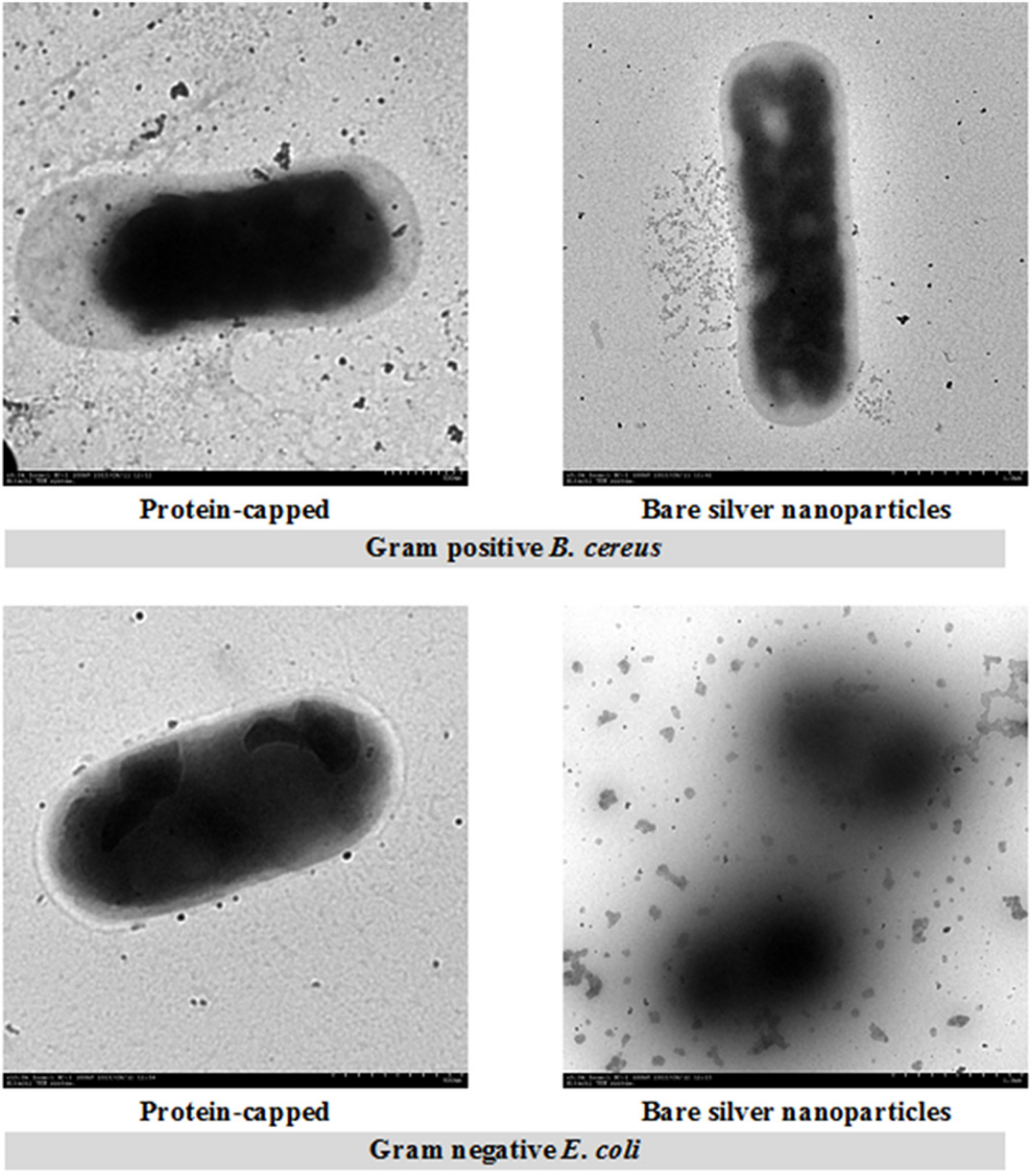
Transmission electron micrographs depicting the morphological changes in Gram positive *B*. *cereus* and Gram negative *E*. *coli* after exposure to protein-capped and bare silver nanoparticles.

TEM measurements were carried out to determine the distribution and location of the silver nanoparticles, as well as morphology of the bacteria after treatment with protein-capped or bare silver nanoparticles. TEM micrographs of representative Gram positive (*B*. *Cereus*) and Gram negative (*E*. *coli*) bacterium showed that protein-capped silver nanoparticles resulted in lesser membrane damage. In contrast, bare silver nanoparticles caused the complete loss of membrane integrity and resulted in the release of cytoplasm (Fig 12).

## Conclusions

The present study demonstrates an economical and environmentally affable approach for the synthesis of protein-capped silver nanoparticles. The extracellular nature of synthesis makes the present method simple, straightforward and allows ease in downstream processing. The as-synthesized nanoparticles were spherical, mono-dispersed and coated with protein molecules. To the best of our knowledge, the present investigation first time demonstrates that the presence of capping molecules significantly influences the activity of biologically synthesized nanoparticles. Removal of capping molecules (protein shell) from the surface of silver nanoparticles showed significant increase in their antibacterial activity against both Gram-positive and Gram-negative bacterial species. The present findings strongly postulate that surface modifications of nanoparticles with proteins can significantly modulate their functional properties.

## Supporting Information

S1 Fig UV-visible absorption spectrum of supernatant showing presence of proteins. (TIF)

S2 Fig Growth dynamics of tested bacterial species in presence of protein-capped or bare silver nanoparticles as compared to controls. (TIF)

S1 Table Magnitude of membrane leakage from bacterial cells after exposure to silver nanoparticles. (DOC)
