# Supplementary figures and images for: Removal of Protein Capping Enhances the Antibacterial Efficiency of Biosynthesized Silver Nanoparticles
Source: PLoS One. 2015 Jul 30;10(7):e0134337. doi: 10.1371/journal.pone.0134337 (PMC4520467; doi:10.1371/journal.pone.0134337)

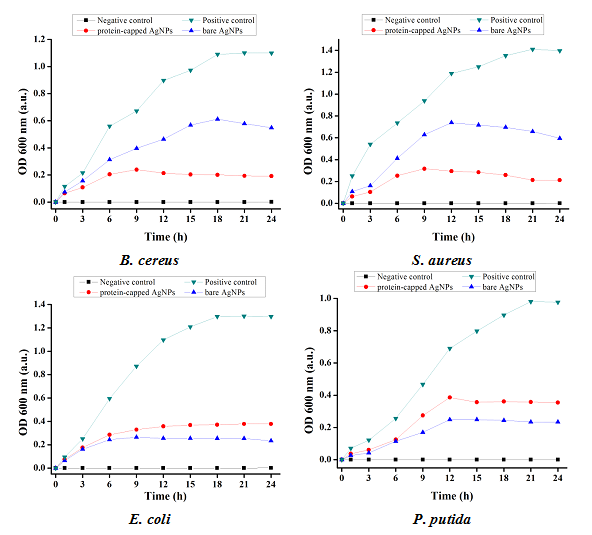

Supplement: S2 Fig — (TIF) [file pone.0134337.s002.tif]
