# Supplementary material for: Removal of Protein Capping Enhances the Antibacterial Efficiency of Biosynthesized Silver Nanoparticles
Source: PLoS One. 2015 Jul 30;10(7):e0134337. doi: 10.1371/journal.pone.0134337 (PMC4520467; doi:10.1371/journal.pone.0134337)
Supplement: S1 Table — (DOC) [file pone.0134337.s003.doc]

***S1 Table*** *Magnitude of membrane leakage from bacterial cells after exposure to silver nanoparticles.*

| **Bacteria** | **Protein content (µg ml^-1^)** | | | **Carbohydrate content (µg ml^-1^)** | | | **Nucleic acid content (µg ml^-1^)** | | |
| --- | --- | --- | --- | --- | --- | --- | --- | --- | --- |
|  | **Control** | **Protein-capped silver nanoparticles** | **Bare silver nanoparticles** | **Control** | **Protein-capped silver nanoparticles** | **Bare silver nanoparticles** | **Control** | **Protein-capped silver nanoparticles** | **Bare silver nanoparticles** |
| **Gram positive** |  |  |  |  |  |  |  |  |  |
| *B. cereus* | 3.99 ± 0.1 | 99.54 ± 9.4 | 108.33 ± 14.7 | 2.19 ± 0.5 | 88.13 ± 3.5 | 97.28 ± 6.3 | 0.98 ± 0.09 | 7.74 ± 0.5 | 9.43 ± 0.6 |
| *S. aureus* | 4.78 ± 0.1 | 88.34 ± 14.9 | 94.86 ± 9.6 | 6.42 ± 1.0 | 93.86 ± 5.3 | 105.47 ± 9.9 | 0.75 ± 0.13 | 8.73 ± 0.4 | 8.92 ± 0.3 |
| **Gram negative** |  |  |  |  |  |  |  |  |  |
| *E. coli* | 8.33 ± 0.6 | 134.38 ± 9. 6 | 154.72 ± 12.2 | 6.28 ± 1.3 | 128.41 ± 9.9 | 144.49 ± 5.4 | 0.35 ± 0.07 | 10.45 ± 0.2 | 11.73 ± 0.7 |
| *P. putida* | 6.54 ± 0.3 | 105.85 ± 6.5 | 129.26 ± 14.3 | 3.57 ± 0.8 | 113.37 ± 8.1 | 128.46 ± 9.8 | 0.57 ± 0.19 | 10.72 ± 0.4 | 14.37 ± 0.2 |

± represents standard error (n = 3)
